# Acquired Gerbode Defect in a Patient with *Staphylococcus Lugdunensis* Aortic Valve Endocarditis

**DOI:** 10.1016/j.case.2021.01.006

**Published:** 2021-03-04

**Authors:** Edward Nabet, Beevash Ray

**Affiliations:** NYU Winthrop Hospital, Cardiology Department, Mineola, New York

**Keywords:** Ventricular septal defect, Gerbode endocarditis

## Abstract

• We diagnosed a rare complication of aortic valve endocarditis.
• *Staphylococcus lugdunensis* has shown to be a destructive bacterium.
• Variations of ventricular septal defects are explored.
• Workup and management of Gerbode defects are described.

## Graphical abstract

**Figure:**
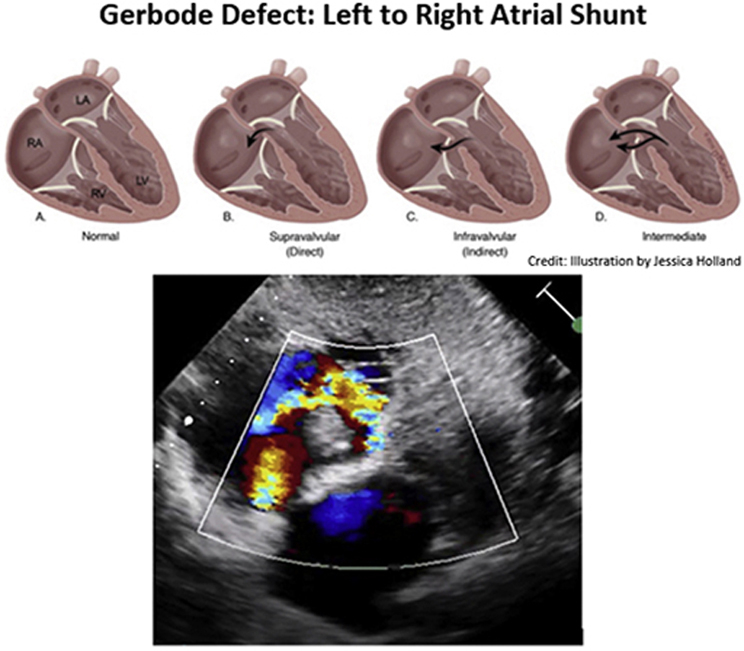

## Introduction

Left ventricle to right atrial shunt, a Gerbode defect, is an extremely rare complication of infective endocarditis. This ventricular septal defect is named after Frank Gerbode, who successfully performed surgery on five patients with this anomaly in 1958.^1^ This rare defect accounts for only 0.08% of intracardiac shunts^2^ and <1% of all congenital cardiac defects.^3^

Endocarditis can lead to destruction of the interventricular septum, causing an acquired Gerbode defect. *Staphylococcus lugdunensis* (*S. lugdunesis*) is a virulent coagulase-negative staphylococcus that can cause rapidly progressive endocarditis with valve and septal destruction. This organism accounts for around 1.1% of all cases of infective endocarditis.^4^ This bacterium was first identified as a causal pathogen of infective endocarditis in 1988 by Freney *et al.*^5^ *S. lugdunensis* endocarditis often requires immediate and aggressive treatment including surgical intervention.^6^

We report a case of *S. lugdunesis* aortic valve endocarditis that was complicated by an acquired Gerbode defect. Our case will highlight how antibiotic therapy alone may lead to treatment failure and acute decompensation.

## Case Presentation

A 66-year-old Caucasian male presented with generalized weakness, followed by a near syncopal episode. The patient had been discharged from our hospital 60 days prior to presentation after being treated for *S. lugdunesis* bacteremia. Blood cultures on prior admission showed that the organism was resistant only to penicillin and was susceptible to all other cephalosporins. Magnetic resonance imaging of the brain showed multiple small foci of recent ischemic damage within the infratentorial and supratentorial compartments concerning for a cardioembolic source. Transesophageal echocardiogram on prior admission had shown severe aortic calcification and thickening, mild to moderate aortic stenosis, mild aortic regurgitation, and no definitive vegetation (Videos 1 and 2). He was discharged home and completed a 6-week course of cefazolin 2 g intravenous every 8 hours for presumed *S. lugdunensis* endocarditis. Blood cultures cleared within 3 days of antibiotic therapy. Symptoms including fevers, chills, and gait disturbance had subsided. There was no follow-up echocardiogram performed prior to readmission.

The review of symptoms on this presentation was positive for dizziness and lower extremity edema. He denied fevers, chills, weight loss, vision changes, focal weakness, sensory disturbance, chest pain, shortness of breath, or palpitations. Past medical history was significant for peripheral arterial disease, tobacco abuse, nonobstructive coronary artery disease, hyperlipidemia, chronic obstructive pulmonary disease, and type II diabetes mellitus. His vital signs on presentation included heart rate, 100 beats per minute; blood pressure, 102/53 mm Hg; respiratory rate 30 breaths per min; oxygen saturation, 83% on room air; and temperature, 99.1 °F. Physical exam revealed a 3/6 pansystolic murmur at the left sternal border and a 3/6 early decrescendo diastolic murmur at the left lower sternal border, bibasilar rales, and 2+ pitting lower extremity edema to the knees bilaterally with cool distal extremities throughout. The remaining physical exam was benign. Labs showed a metabolic acidosis, leukocytosis, thrombocytopenia, acute kidney injury, transaminitis, elevated troponin, venous blood gas pH 7.15, PCo2 32 mm Hg, PO2 24 mm Hg, bicarbinate 11 mEq/L, lactate 15.4 mmol/L, B-type natriuretic peptide 812 pg/mL, and troponin 1.3 ng/mL. COVID-19 polymerase chain reaction testing was negative.

Two sets of blood cultures were positive for *S. lugdunensis.* The bacteria had an identical resistance profile when compared with the organism from his first admission.

**Figure 1:**
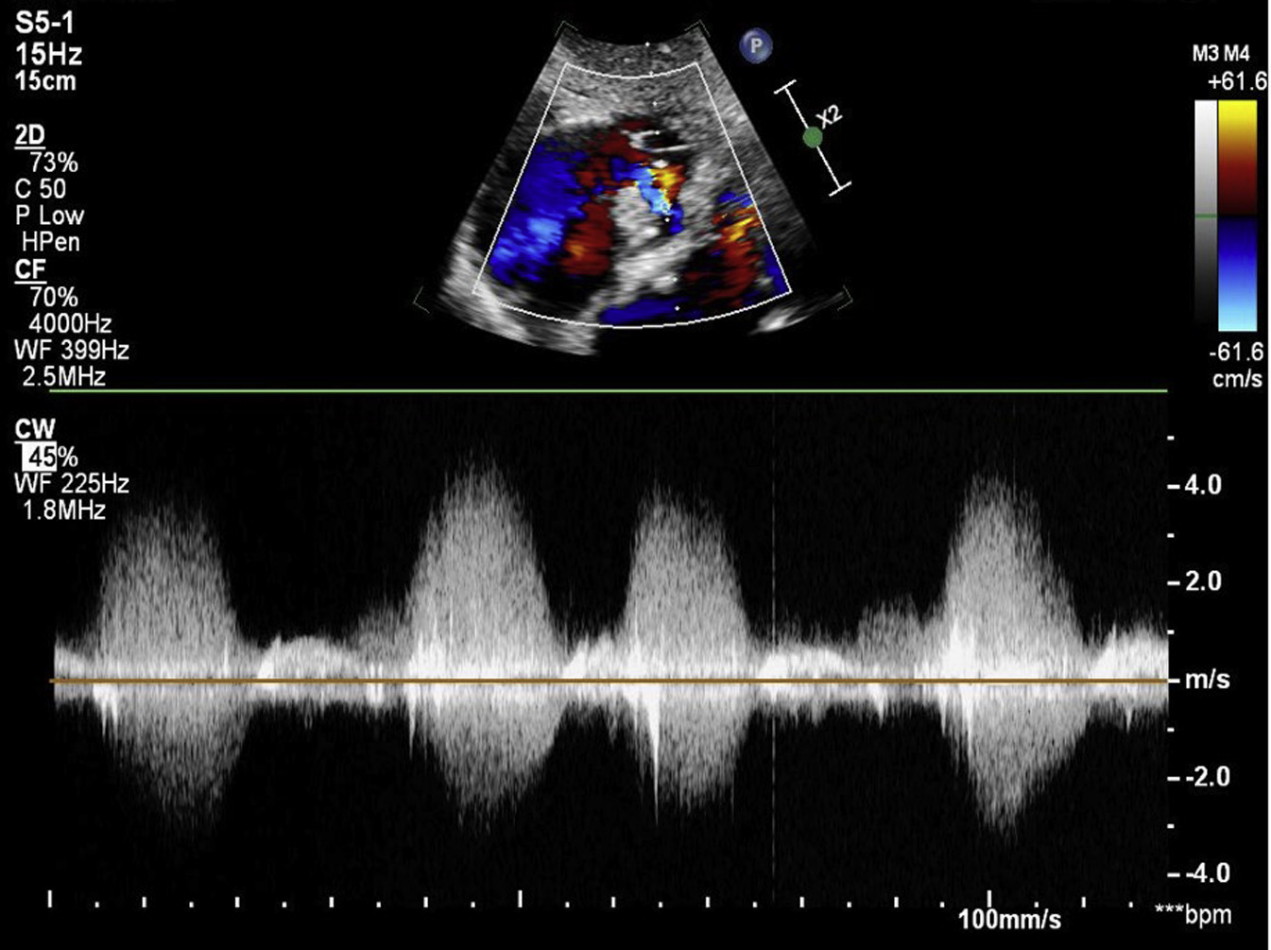
Continuous-wave Doppler through Gerbode defect. Peak velocity is 4.7 m/sec.

Transthoracic echocardiogram revealed mildly reduced left ventricular systolic function, ejection fraction 40%, severely calcified aortic valve, severe aortic regurgitation with a pressure half time of 114 msec (Videos 3 and 4). Echocardiogram also showed a 1.5 × 0.8 cm mobile echodensity in the right atrium attached to a nonmobile calcified appearing mass measuring 2.3 × 1.5 cm adjacent to the coronary sinus inflow, and high-velocity flow from the left ventricle to right atrium, which was consistent with a Gerbode defect (Videos 5 and 6). The peak velocity of the flow through the ventricular septal defect was measured at 4.7 m/sec (Figure 1). There was mild right atrial dilatation. The right ventricular systolic function was normal.

The mobile mass within the right atrium appeared to represent thrombus versus vegetation. The right atrial echodensity and Gerbode defect were not apparent on echocardiogram 2 months prior. The aortic regurgitation appeared worse. Once again, transthoracic echocardiogram could not rule out vegetation on the aortic valve given its severe calcification and thickening.

Electrocardiogram showed normal sinus rhythm, left-axis deviation, voltage criteria for left ventricular hypertrophy, and anterior asymmetric T-wave inversions without ST changes. Transesophageal echocardiogram was deferred given concerns for disseminated intravascular coagulation.

Repeat labs showed persistent lactic acidosis respite fluid resuscitation. Labs also were concerning for disseminated intravascular coagulation with platelets downtrending to 16,000, hemoglobin to 7.0 g/dL, and low haptoglobin and fibrinogen.

The patient was then started on vancomycin 1,250 mg intravenous every 12 hours, meropenem 500 mg intravenous every 8 hours, and norepinephrine 0.8 µg/min intravenous for septic shock thought to be secondary to recurrent *S. lugdunensis* endocarditis. Despite intravenous vasopressors, he remained hemodynamically unstable with signs of refractory shock. On day 2 of his admission the patient went into cardiac arrest due to severe metabolic derangements. He was unable to be resuscitated prior to emergent surgical intervention.

## Discussion

Gerbode defects can be congenital or acquired. The congenital form accounts for <1% of all congenital heart disease and was first classified by Gerbode.^7^ Acquired defects from endocarditis are even less common. Sinisalo *et al.*^8^ reported 65 cases of acquired origin in a thorough review of the literature from 1865 to 2009. Gerbode defect, a left ventricle to right atrial shunt, has two anatomical variants. Indirect defects involve flow through the septal leaflet of the tricuspid valve and are considered infravalvular.^3^ Direct defects cross the membranous septum above the tricuspid valve and are termed supravalvular. The tricuspid valve usually has a slight apical displacement when compared to the mitral valve, which creates the opportunity for this defect to form.

*S. lugdunensis* is a virulent coagulase-negative staphylococcus that is rarely encountered. It has a tendency to involve left heart valves and be more destructive and rapidly progressive when compared to other coagulase-negative staphylococcal species.^4^ Unlike other coagulase-negative staph, *S. lugdunensis* has Fbl, a fibrinogen-binding protein with similar properties to clumping factor A, which is a fibrinogen-binding protein of *Staphylococcus aureus* that plays an important role in its virulence.^9^

Endocarditis secondary to coagulase-negative *S. lugdunensis* is a rare clinical entity that has significant clinical importance. We report a case of recurrent *S. lugdunensis* aortic valve endocarditis complicated by Gerbode defect, severe aortic regurgitation, and possible right atrial thrombus formation. Other cases of *S. lugdunensis* endocarditis complicated by ventricular septal defects have been reported. However, this is the first case report to show the combination of aortic valve involvement with left ventricular to right atrial shunting and possible right atrial thrombus versus vegetation formation.

Sabe *et al.*^10^ reviewed 13 cases of *S. lugdunensis* endocarditis from 2002 to 2011 at their institution. They found that 10 cases had bulky vegetations, three had abscess formation, and four had perforation. Fifty percent of the cases had predisposing structural valvar heart disease; five cases were treated successfully with surgery; and one death was seen in a medically managed patient

A literature review completed in 2015 by Prifti *et al*. found 25 previously published cases with acquired Gerbode defect without prior cardiac surgery, 21 of which were caused by endocarditis. Eleven of these cases involved the aortic valve, and only one case was caused by *S. lugdunensis*. Nineteen cases of endocarditis were treated with surgery, and two conservatively. The mortality was almost 9% in patients with endocarditis. The overall mortality in 26 patients without prior cardiac surgery was 15.4%. Complete atrioventricular block is a complication that was seen in 13% of these patients.^11^

When making the diagnosis of Gerbode defect, one would expect high-velocity systolic Doppler from the left ventricle to right atrium due to the large pressure gradient. The peak velocity recorded for our Gerbode defect was 4.7 m/sec. When interpreting the Doppler signals, physicians must not inaccurately identify these high-velocity signals as tricuspid regurgitation. When more accurate visualization of the defect is needed, transesophageal echocardiogram should be used.

Treatment of acquired Gerbode defect depends on symptoms, severity of the shunt, accompanying valve abnormalities, and comorbidities. The majority of cases require surgical intervention.^11^

## Conclusion

*S. lugdunensis* is a highly destructive organism that should be approached in a similar way as *Staphylococcus aureus* in regards to bacteremia and endocarditis. Transesophageal echocardiogram as well as cardiothoracic surgery evaluation should not be delayed in patients presenting with *S. lugdunensis* bacteremia. Persistent infection may cause rapidly progressive endocarditis with valve and septal destruction, which includes the ever rare Gerbode defect.

## Supplementary Data

Video 1 Transesophageal echocardiogram 2 months prior to presentation. Midesophageal aortic valve short-axis (*left*) and long-axis (*right*) views. Severely calcified aortic valve with mild to moderate aortic stenosis, mild aortic regurgitation, and aortic valve area of 1.7 cm^2^ by two-dimensional planimetry.

Video 2 Transesophageal echocardiogram 2 months prior to presentation. Midesophageal aortic valve short axis in three dimensions. Severe calcification and thickening of aortic valve and no mobile vegetation. Cannot rule out endocarditis.

Video 3 Transthoracic echocardiogram long-axis view. Severely calcified aortic valve with moderate to severe aortic regurgitation.

Video 4 Transthoracic echocardiogram apical five-chamber view. Severely calcified aortic valve with severe aortic regurgitation.

Video 5 Transthoracic echocardiogram subcostal view: transthoracic echocardiogram 1.5 × 0.8 cm mobile echodensity in the right atrium, attached to a nonmobile, calcified appearing mass measuring 2.3 × 1.5 cm. The right atrial mass may represent thrombus or vegetation.

Video 6 Transthoracic echocardiogram subcostal view. High-velocity flow entering the right atrium adjacent to the interatrial septum/left ventricular junction.
